# Dictionary for the Blind

**Published:** 1860-11

**Authors:** 


DICTIONARY FOR THE BLIND.—A Dictionary of the English language for the use of the Blind has just been published in this city, in raised letters, in three folio volumes, of two hundred pages each. The author is Professor Dunglison, in conjunction with Mr. William Chapin, the one the Chairman of the Committee of Instruction, the other the Principal of the Pennsylvania Institution for the Instruction of the Blind, one of those noble establishments which abound in Philadelphia for the amelioration of the mental and moral condition of the races. The work is based upon the comprehensive Dictionary of Worcester, and is the first book of the kind ever published. Some estimate of the labors of Professor Dunglison may be formed when it is stated that he bestowed upon the work not less than four years. Much as our illustrious countryman has written, posterity will not regard the Dictionary for the use of the Blind as among the least valuable of his publications.

